# Elevated E2F6 Expression in Colorectal Cancer Tissues and Its Association With Clinicopathological Features

**DOI:** 10.14740/wjon2707

**Published:** 2026-05-08

**Authors:** Da Tong Zeng, Ke Jun Wu, Jian Di Li, Guo Qiang Chen, Wei Zhang, Zong Yu Li, Jing Wen Ling, Wei Jian Huang, Gang Chen, Hui Li

**Affiliations:** a Department of Pathology, The First Affiliated Hospital of Guangxi Medical University, Nanning 530021, Guangxi Zhuang Autonomous Region, China; b Department of Pathology, Redcross Hospital of Yulin City, Yulin 537000, Guangxi Zhuang Autonomous Region, China; c Department of Computer Science and Technology, School of Computer and Electronic Information, Guangxi University, Nanning 530004, Guangxi Zhuang Autonomous Region, China; d Department of Colorectal and Anal Surgery, The First Affiliated Hospital of Guangxi Medical University, Nanning 530021, Guangxi Zhuang Autonomous Region, China; e These authors contributed equally to this article.

**Keywords:** Colorectal cancer, E2F transcription factor 6, Clustered regularly interspaced short palindromic repeats, Spatial transcriptomics, Clinicopathological features, Diagnostic biomarker, Tumor budding

## Abstract

**Background:**

Colorectal cancer (CRC) is the third most common malignancy worldwide, and the role of E2F transcription factor 6 (E2F6) in CRC remains controversial.

**Methods:**

We analyzed E2F6 mRNA expression across 19 platforms (2,449 CRC patients and 1,328 controls), evaluated expression patterns using single-cell RNA sequencing (scRNA-seq) and spatial transcriptomics, assessed E2F6 dependency using clustered regularly interspaced short palindromic repeats (CRISPR) knockout data from 52 CRC cell lines, and validated protein expression by immunohistochemistry (IHC) in 200 paired CRC and adjacent tissues. Associations between E2F6 and clinicopathological features were analyzed.

**Results:**

E2F6 was significantly upregulated in CRC versus controls (summary receiver operating characteristic (sROC) area under the curve (AUC) = 0.93), supported by scRNA-seq and spatial transcriptomics. E2F6 knockout suppressed proliferation across CRC cell lines, and IHC confirmed higher E2F6 protein expression (AUC = 0.91). Elevated E2F6 correlated with adverse clinicopathological features including female sex, age ≥ 60 years, advanced T stage, high-grade tumor budding, and higher histological grade.

**Conclusions:**

E2F6 is highly expressed in CRC and is associated with unfavorable clinicopathological features, supporting its potential utility as a diagnostic biomarker and a candidate target for CRC stratification and therapy development.

## Introduction

In 2020, over 1.9 million new colorectal cancer (CRC) cases and 930,000 deaths were estimated from the GLOBOCAN database. Incidence and mortality rates varied by region around the world. By 2040, the burden may reach 3.2 million cases and 1.6 million deaths, which leads to its third common cancer worldwide [1–3]. The pathogenesis of CRC is intricate, involving a myriad of genetic, environmental, and lifestyle factors [4–7]. Key mechanisms contributing to its development include the adenoma-carcinoma sequence, the serrated pathway, chromosomal instability, and microsatellite instability, all of which are closely linked to mutations in critical genes such as *APC, TP53, RAS, BRAF, HER2,* and *PTEN* [8, 9]. The heterogeneity of CRC further complicates early detection and effective treatment strategies [10, 11]. While current interventions, including screening and surgical options, have led to improved outcomes [12], the prognosis for advanced-stage CRC remains grim due to the complex and not fully understood molecular mechanisms of its pathogenesis.

Recently, there has been an increasing emphasis on elucidating the molecular mechanisms underlying the progression of CRC. Preliminary research has been conducted on the expression and mechanisms of certain members of the E2F family in CRC [13–18]. Among the members of the E2F family, sporadic reports have emerged regarding the significance of E2F6 expression and its superficially understood modes of action in CRC [19, 20]. Transcriptional levels of E2F6 in colon cancer patients were solely examined using data from The Cancer Genome Atlas (TCGA) and Genotype-Tissue Expression (GTEx) [20]. As Yao et al reported, no significant difference in E2F6 expression levels was found between colon cancer and non-CRC tissues [20]. However, this study had a single detection method, relying only on RNA-seq, and its conclusion was based on data from a single research center, which might introduce biases. Another study utilized reverse transcription quantitative polymerase chain reaction (RT-qPCR) to detect E2F6 in 46 CRC samples and found an up-regulation of E2F6 mRNA in CRC [19], which was completely contrary to the results reported by Yao et al [18]. Similarly, this study also employed only one detection method and had a sample size of less than 50 [19]. Therefore, it is currently essential to integrate various means of detecting E2F6 to comprehensively evaluate E2F6’s role in CRC progression.

Therefore, this research first demonstrated the changes in CRC cell growth following the knockdown of E2F6 by clustered regularly interspaced short palindromic repeats (CRISPR) screening at the cell line level. Subsequently, it conducted multi-level investigations into the expression levels of E2F6 mRNA at the CRC tissue and single-cell levels. Additionally, clinical samples were utilized for inhouse immunohistochemistry (IHC) to display the protein level, and spatial transcriptomics technology was employed to further validate the expression pattern of E2F6 in CRC tissues.

## Materials and Methods

### Single-cell RNA sequencing (scRNA-seq) analysis

scRNA-seq data were downloaded from Gene Expression Omnibus (GEO) (GSE144735). Seurat version 5.0.0 package was utilized for quality control, t-distributed stochastic neighbor embedding (tSNE) dimension reduction, and cell annotation. The expression level of E2F6 was visualized using density plot. scRNA-seq data were downloaded from GEO (GSM8265213). Seurat version 5.0.0 package was utilized for quality control, dimension reduction, and spatial expression analysis. The expression locations of E2F6, as well as CRC markers (CDX2 and KRT20), were visualized in a slide.

### Process of collecting and processing datasets

We collected the expression profiles of E2F6 in CRC from several key sources, including the Gene GEO, TCGA, GTEx project, the International Cancer Genome Consortium (ICGC), ArrayExpress, the Sequence Read Archive (SRA), and pertinent scientific literature. The search keywords included “colon,” “rectal,” “rectum,” “colorectal,” “carcinoma,” “cancer,” “malignancy,” and “tumor.” The inclusion criteria were as follows: 1) data derived from human samples; 2) experimental groups consisting of CRC tissue samples, with control groups comprising non-tumor tissues; 3) a minimum of three samples in each group. The exclusion criteria were: 1) samples from recurrent or metastatic CRC; 2) absence of E2F6 expression data; 3) fewer than three samples in any group. Following the screening process, datasets from the same GEO platform were consolidated to form a comprehensive expression matrix. The mRNA expression matrix was standardized and log_2_(x + 1) transformed, with batch effects removed using the “limma” and “sva” packages in R.

### Examination of E2F6 transcript levels in CRC lines

The transcriptional abundance of E2F6 within CRC cell lines was assessed utilizing the mRNA expression data obtained from the Cancer Cell Line Encyclopedia (CCLE). The mRNA expression analysis was conducted employing R software, version 4.0.3, and the results were graphically represented using horizontal bar charts with the aid of ggplot2, version 3.3.3 [21].

### Investigation of the role of E2F6 in controlling the proliferation of CRC cell

The functionality of E2F6 within CRC cells was investigated using CRISPR-based knockout screening. The importance of E2F6 in a range of CRC cell lines was determined by computing dependency scores through the CERES algorithm. A dependency score that is negative, signifying reduced cell growth post-E2F6 knockout, implies that E2F6 is crucial for proliferation. Conversely, a positive dependency score, indicating improved growth upon E2F6 knockout, suggests that E2F6 may act as an inhibitor in these cell lines [22].

### Inhouse IHC validation of E2F6 protein expression in CRC

This study utilized a tissue microarray containing 200 pairs of CRC and corresponding non-tumor tissue samples from the Red Cross Hospital in Yulin, Guangxi, to elucidate the expression of E2F6 protein in CRC. We collected clinical pathological data from the patients, including age, gender, macroscopic subtype, vascular invasion, neural invasion, lymph node involvement, tumor-node-metastasis (TNM) staging, survival status, and clinical stage. Ethical approval for this study was obtained from the Ethics Committees of both the Red Cross Hospital in Yulin and the First Affiliated Hospital of Guangxi Medical University (No. Z20210442). All participants provided written informed consent to participate in this study. This study was conducted in accordance with the Declaration of Helsinki.

Tissue microarrays and IHC were prepared and analyzed in strict accordance with the relevant manufacturer’s standards of workmanship and the procedures and results were checked and qualified by a pathologist. The number of positive cells was assessed by randomly counting 100 positive cells in each field of view at the highest magnification. Two experienced pathologists independently evaluated the processed sections. The evaluation criteria included the percentage of positively stained cells and staining intensity. The scoring system for the percentage of positively stained cells was as follows: 0 points for no expression, 1 point for fewer than 25% positive cells, 2 points for 26% to 50% positive cells, 3 points for 51% to 75% positive cells, and 4 points for over 75% positive cells. The staining intensity was scored as 0 for unstained, 1 for weak cytoplasmic staining, 2 for moderate yellow staining, and 3 for strong brown-yellow staining [23–25]. The overall score was derived by multiplying the staining intensity by the percentage of positively stained cells. In cases where the scores from the two pathologists differed, the average of their scores was taken as the final score.

### Statistical analysis of E2F6 expression in CRC

The E2F6 protein’s differential expression in CRC tissues and non-CRC colorectal tissues was examined using Student’s *t*-test via IBM SPSS Statistics 26 software. Violin plot and receiver operating characteristic (ROC) curves were generated using GraphPad Prism 8 software.

The ROC curves were employed to evaluate E2F6’s capability to discriminate between CRC tissues and non-CRC colorectal tissues, with the area under the curve (AUC) serving as a measure of its accuracy. Unpaired two independent samples *t*-test was employed to analyze enumeration data, specifically investigating the associations between E2F6 expression and various clinicopathological parameters, including sex, age, TNM stage, pathological stage, pathological type, tumor size, tumor number, nerve invasion, and vascular invasion. These parameters were obtained from IHC samples. Statistical significance was assessed at a threshold of P < 0.05.

The expression levels of E2F6 in 200 patients from the Yulin Red Cross Hospital were analyzed using GraphPad Prism 8.0.2 for their Pearson correlations with the Ki-67 positivity rate, histological type, and pathological grade. Additionally, Kaplan-Meier survival analysis was conducted on the high and low expression groups of E2F6 in the aforementioned cases.

## Results

### scRNA-seq analysis

tSNE plots were used to identify and distinguish major cell populations in CRC based on transcriptional profiles. Cell type annotation was performed using canonical marker genes (e.g., EPCAM/KRTs for epithelial cells, PTPRC for immune cells, PECAM1/VWF for endothelial cells, and COL1A1/DCN for fibroblasts), and malignant epithelial cells were further distinguished from normal epithelial cells based on established malignancy-related transcriptional features (see “Methods”).

**Figure 1:**
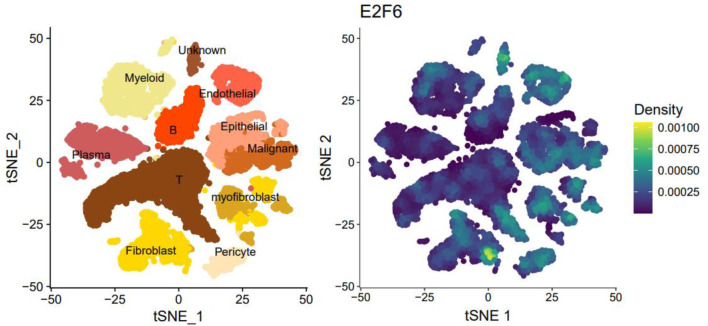
The tSNE plot of single-cell sequencing data and the expression plot of E2F6.

E2F6 showed a higher expression trend in malignant epithelial cells compared with normal epithelial cells (Fig. 1). Notably, E2F6 signal was also observed in additional cell populations beyond malignant cells, particularly in subsets with proliferative features, which is consistent with the known role of E2F family members in cell-cycle regulation. This broader distribution is explicitly shown in Figure 1 and is considered in the biological interpretation of the scRNA-seq results.

### Spatial transcriptomics sequencing (ST-seq)

**Figure 2:**
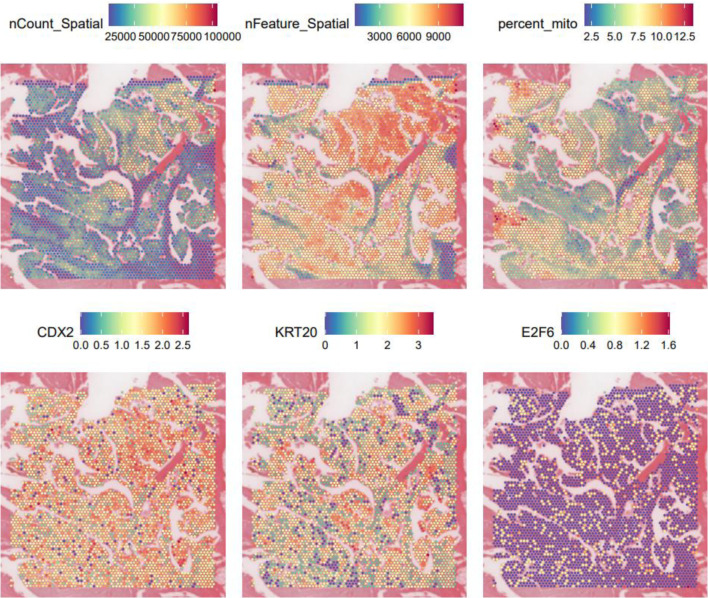
Spatial transcriptome data gene expression level maps and expression maps of E2F6, CDX2, and KRT20.

ST-seq was used to evaluate the *in situ* distribution of E2F6 expression across the CRC tissue section. E2F6 displayed heterogeneous expression across spatial spots within the CRC slide (Fig. 2). To clarify the biological implication of this heterogeneity, we examined the spatial co-localization of E2F6 with epithelial differentiation markers CDX2 and KRT20. CDX2 and KRT20 were included because they are widely used markers of colorectal epithelial identity and differentiation, enabling interpretation of whether E2F6-enriched regions align with epithelial tumor compartments.

Consistent with the scRNA-seq findings, spatially elevated E2F6 tended to localize to epithelial/tumor-enriched areas, while lower expression was observed in other regions, supporting a tissue-contextualized pattern rather than uniform upregulation across the section. These results suggest that E2F6 may mark specific tumor micro-regions with distinct transcriptional states.

### Enhanced expression of E2F6 mRNA in CRC

#### Assessment of extensive datasets derived from microarray and sequencing studies worldwide

Figure 1 illustrates the stringent selection criteria applied to the E2F6 mRNA dataset. This comprehensive analysis spanned across 19 distinct platforms. The study encompassed a substantial number of subjects, with a total of 2,449 individuals diagnosed with CRC and 1,328 controls, thereby allowing for an in-depth evaluation of E2F6 mRNA levels in the context of CRC.

#### Enhanced E2F6 mRNA expression in CRC

**Figure 3:**
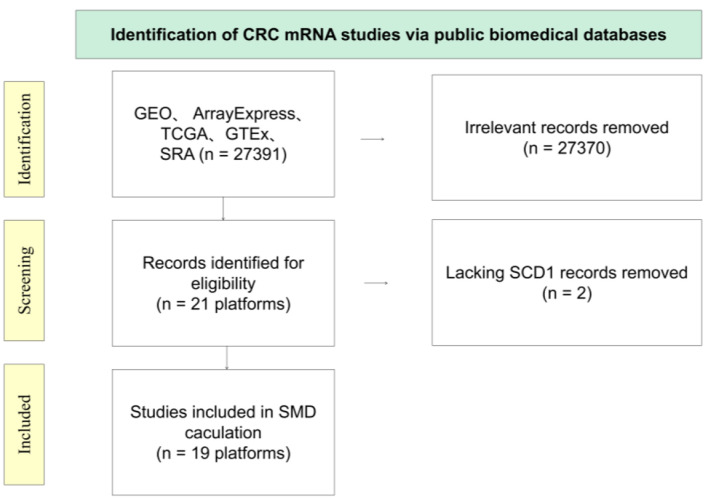
Diagrammatic overview of the E2F6 mRNA dataset screening process.

**Figure 4:**
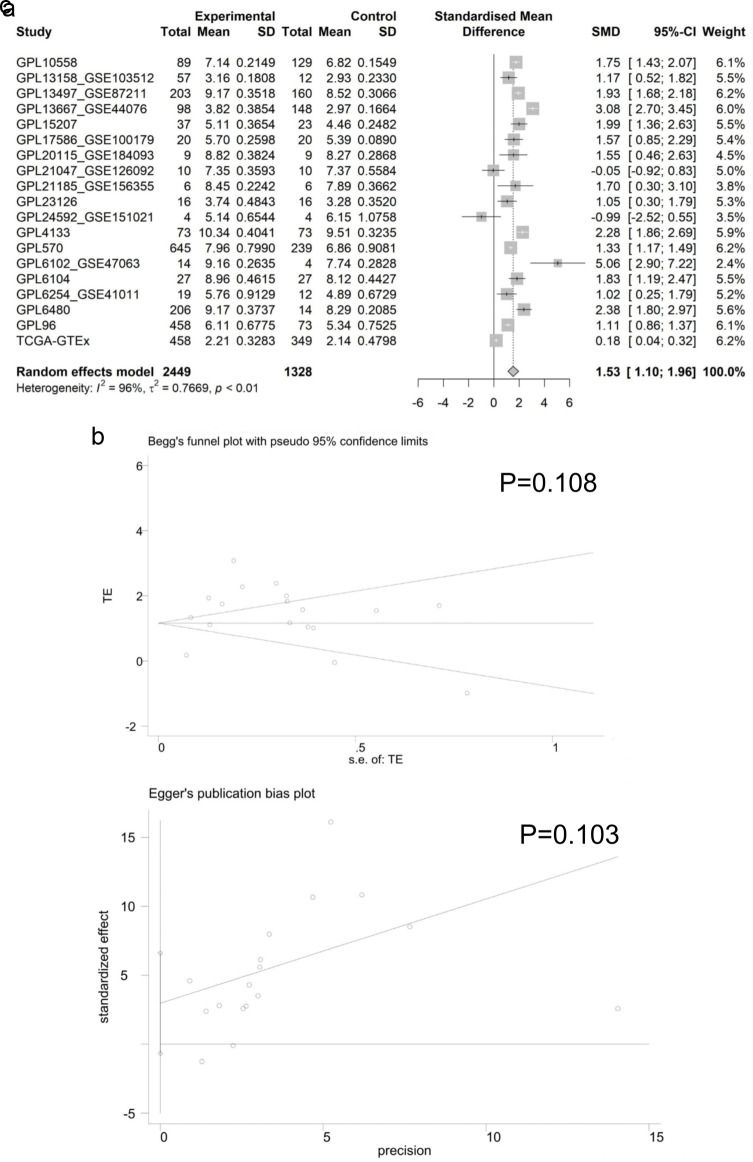
Analysis of E2F6 mRNA Expression in CRC. (a) Forest plot showing the increased E2F6 mRNA levels in CRC compared to non-CRC tissue. (b, c) Results of Egger’s and Begg’s tests for the assessment of publication bias.

Employing the Wilcoxon test to assess differences in E2F6 mRNA levels between CRC and non-CRC tissues, our results indicated a significant increase in E2F6 mRNA in CRC samples (Fig. 3). A comprehensive analysis of data from various studies, using a random effects model, confirmed this upregulation with a standardized mean difference (SMD) of 1.53, a 95% confidence interval (CI) from 1.10 to 1.96, and high heterogeneity (I^2^ = 96%), which was statistically significant (P < 0.01) as illustrated in Figure 4a. Furthermore, Begg’s and Egger’s tests did not indicate any evidence of publication bias, with P-values of 0.108 and 0.103, respectively, as shown in Figure 4b, c.

#### Summary receiver operating characteristic (sROC) analysis of E2F6 expression in CRC

**Figure 5:**
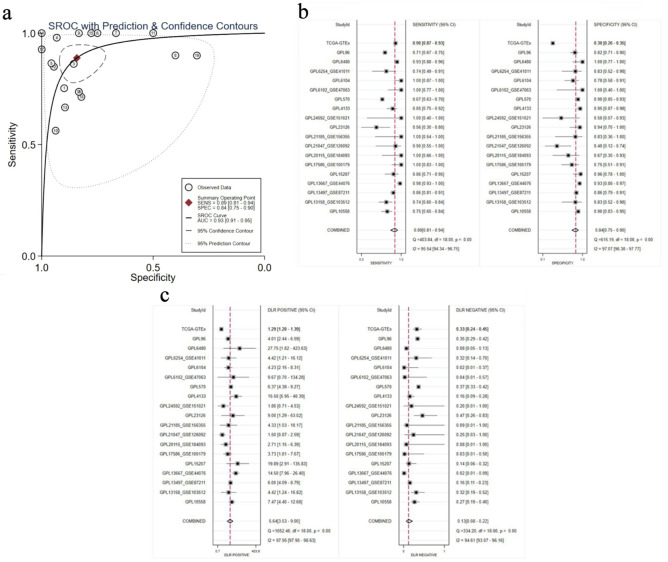
Comprehensive evaluation of the association of E2F6 with CRC outcomes. (a) sROC curve. (b) Sensitivity and specificity. (c) Positive and negative likelihood ratios.

The capacity of E2F6 to distinguish between CRC tissues and non-CRC tissues was evaluated using the sROC curve analysis. The analysis revealed that the AUC for the sROC was 0.93 (95% CI: 0.91–0.95). Additionally, the sensitivity was recorded at 0.89 (95% CI: 0.81–0.94) and specificity was 0.84 (95% CI: 0.75–0.90), as shown in Figure 5a, b. The positive likelihood ratio associated with E2F6 expression was 5.64 (95% CI: 3.53–9.00, I^2^ = 97.95%), and the negative likelihood ratio was 0.13 (95% CI: 0.08–0.22, I^2^ = 94.61%), as illustrated in Figure 5c. Both the sROC curve and the likelihood ratio analysis highlight the significant role of E2F6 in identifying CRC tissues compared to non-CRC.

### Analysis of E2F6 mRNA levels across CRC lines

**Figure 6:**
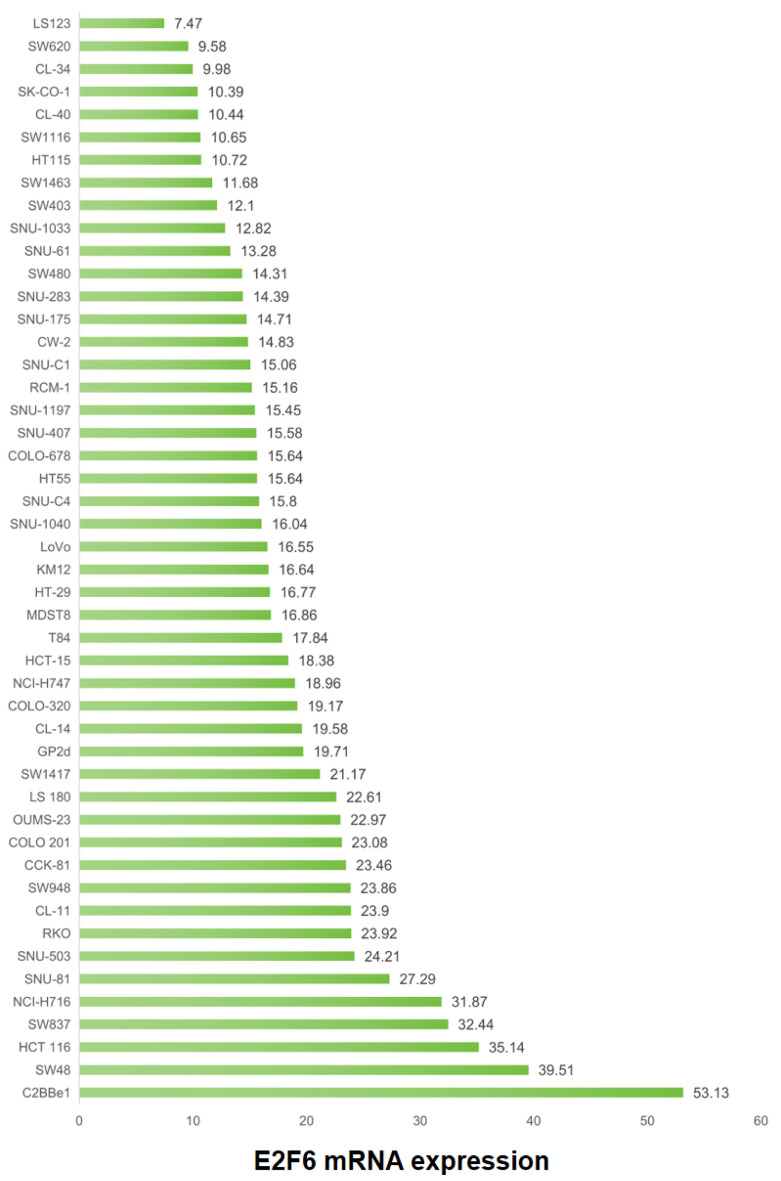
Variability of E2F6 mRNA expression across CRC cell lines (CCLE). Horizontal axis: E2F6 mRNA expression (TPM). Vertical axis: CRC cell line name. Each point represents one cell line. Higher TPM indicates higher transcript abundance.

Figure 6 presents an evaluation of the E2F6 mRNA levels in a panel of 48 CRC cell lines, as obtained from the CCLE database. The analysis indicates a range of E2F6 mRNA expression within these lines, with notable peaks in expression observed in the SW48 and C2BBe1 cell lines. This analysis was included to 1) characterize the baseline variability of E2F6 expression across commonly used CRC models and 2) provide a rationale for selecting representative cell lines for functional interrogation.

### Role of E2F6 in enhancing CRC cell line proliferation

**Figure 7:**
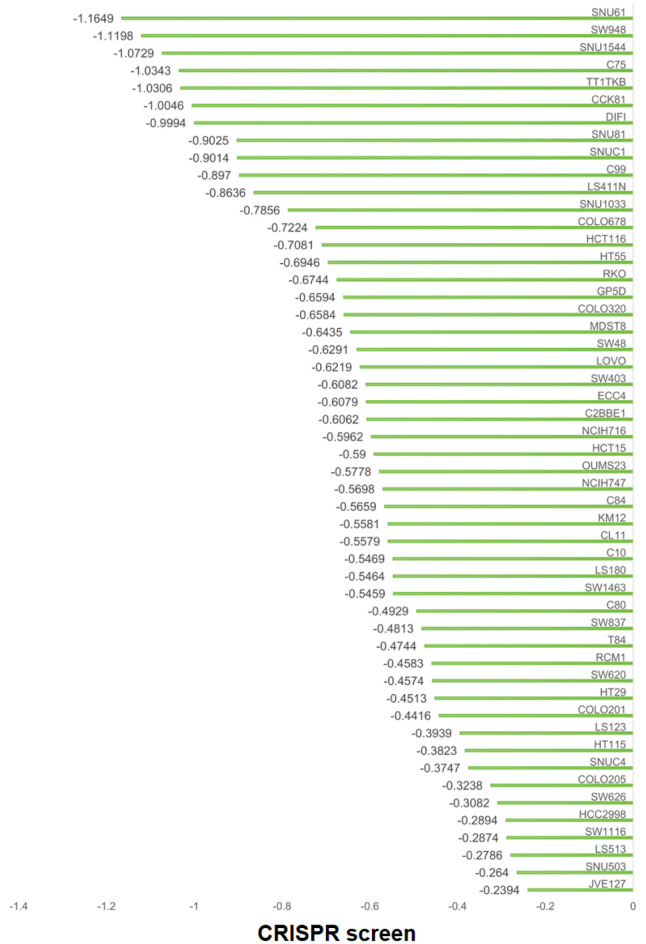
Effects of E2F6 gene deletion on CRC cell line proliferation. Horizontal axis: E2F6 gene effect score for each cell line (CRISPR screen). Vertical axis: CRC cell line name. More negative values indicate reduced proliferation/viability after E2F6 knockout.

CRISPR-based knockout experiments were conducted to explore the E2F6 of contribution to the proliferation of CRC cell lines, as demonstrated in Figure 7. Elimination of the E2F6 gene was associated with a slowdown in proliferation across 52 distinct CRC cell lines, suggesting the involvement of E2F6 in promoting cellular growth in CRC. Specifically, the “gene effect score” reflects the impact of E2F6 deletion on cell viability/proliferation, where more negative scores indicate stronger growth suppression upon gene knockout (i.e., higher dependency on E2F6).

### Comparison of E2F6 protein expression levels in CRC and adjacent non-tumor tissues with inhouse IHC

**Figure 8:**
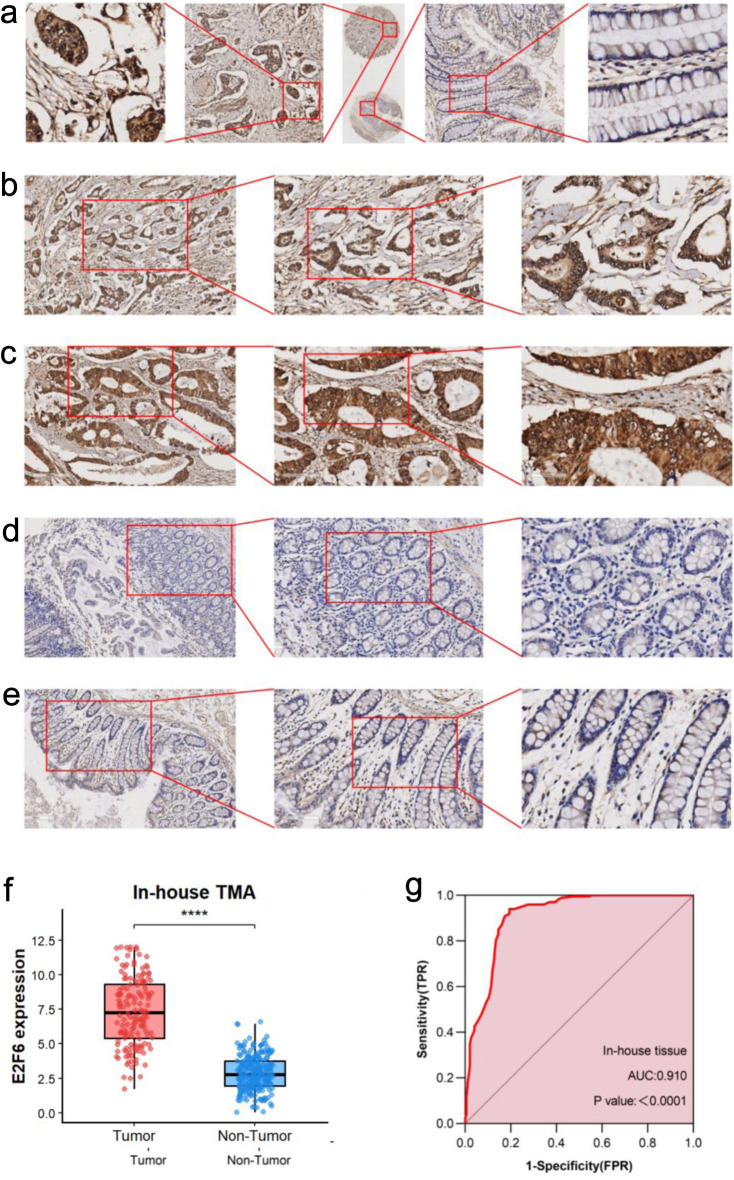
The expression level of E2F6 protein in CRC and peritumor colon tissues based on immunohistochemistry. (a) Representative images of E2F6 protein expression in CRC tissues microarray. In panels, the magnifications of the three images of each panel are 10, 100, and 400 respectively. (b, c) Representative images of E2F6 protein expression in CRC tissues. (d, e) Representative images of E2F6 protein expression in peritumor colon tissues. In panels (b)–(e), the magnifications of the three images of each panel are 100, 200, and 400 respectively. (f) E2F6 protein expression. (g) Receiver operating characteristic (ROC) curve with area under the curve (AUC) of E2F6 protein expression in CRC tissues.

This study assessed the expression of E2F6 protein in CRC tissues and their adjacent non-tumor tissues. Results from IHC analysis of 200 cases demonstrated that the E2F6 staining intensity was significantly greater in CRC tissues compared to adjacent non-tumor tissues (Fig. 8a–e). A quantitative evaluation of E2F6 expression in CRC was performed using violin plots and ROC curves. The violin plot indicated that E2F6 protein expression in CRC tissues was significantly higher than that in the non-cancerous adjacent tissues (P < 0.0001, Fig. 8f). Furthermore, ROC analysis revealed a significant elevation of E2F6 in CRC tissues, with an AUC of 0.910 (Fig. 8g).

### Association of E2F6 expression with clinicopathological parameters in CRC

In the IHC analysis based on internal samples (Table 1), we observed significant differences in E2F6 expression levels with respect to gender, age, T stage, tumor budding, histological type, histological grade, nerve invasion, and lymph node metastasis. No statistically significant differences were found for the remaining clinical parameters.

**Table 1:** Clinical Value of E2F6 Expression in CRC

Parameter	E2F6 high expression	E2F6 low expression	P(t)	t/F
Gender			0.029	1.117
Male	64 (56.1%)	55 (64%)		
Female	50 (43.9%)	31 (36%)		
Age			< 0.001	−2.114
< 60 years	35 (30.7%)	39 (45.3%)		
≥ 60 years	79 (69.3%)	47 (54.7%)		
Location			0.363	0.573
Colon	63 (55.3%)	44 (51.2%)		
Rectum	51 (44.7%)	42 (48.2%)		
T stage			0.001	1.555
Stage I–II	8 (7%)	12 (14%)		
Stage III–IV	106 (93%)	74 (86%)		
Tumor budding			0.026	1.353
BD 1	58 (50.9%)	52 (60.5%)		
BD 2–3	56 (49.1%)	34 (39.5%)		
Clinical stage			0.113	−0.846
Stage I–II	73 (64%)	50 (58.1%)		
Stage III–IV	41 (36%)	36 (41.9%)		
Number of lesions			0.988	−0.008
Single lesion	110 (96.5%)	83 (96.5%)		
≥ 2 lesions	4 (3.5%)	3 (3.5%)		
Macroscopic type			0.036	4.476
Polypoid type	23 (20.2%)	26 (30.2%)		
Ulcerative type	84 (76.7%)	57 (66.3%)		
Infiltrative type	7 (6.1%)	3 (3.5%)		
Histological type			0.114	0.783
Non-special type	89 (78.1%)	71 (82.6%)		
Special type	25 (21.9%)	15 (17.4%)		
Histological grade			0.009	1.306
Low grade	81 (71.1%)	68 (79.1%)		
High grade	33 (28.9%)	18 (20.9%)		
Vascular invasion			0.722	0.178
Yes	21 (18.4%)	15 (17.4%)		
No	93 (81.6%)	71 (82.6%)		
Perineural invasion			0.001	1.649
Yes	16 (14%)	6 (7%)		
No	98 (86%)	80 (93%)		
Lymph node metastasis			0.023	−1.262
Yes	39 (34.2%)	37 (43%)		
No	75 (65.8%)	49 (57%)		
MMR status			0.702	−0.192
dMMR	5 (4.4%)	6 (7%)		
pMMR	96 (84.2%)	74 (86%)		

CRC: colorectal cancer; dMMR: deficient mismatch repair; MMR: mismatch repair; pMMR: proficient mismatch repair.

### Correlation analysis and survival analysis based on inhouse sample IHC results

Pearson correlation analysis revealed a negative correlation between E2F6 expression levels and the Ki-67 positivity rate, while E2F6 expression levels exhibited positive correlations with histological type and pathological grade.

**Figure 9:**
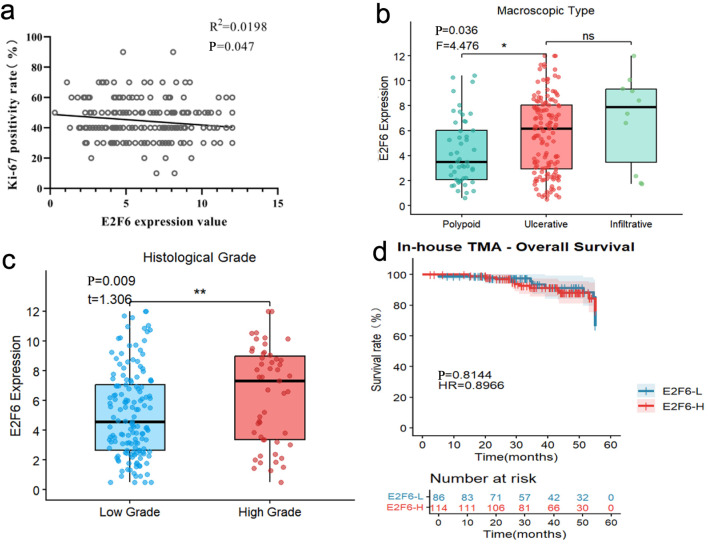
Pearson correlation analysis and Kaplan-Meier survival analysis based on internal sample IHC results. (a) correlation analysis of E2F6 expression value and Ki-67 positivity rate (%). (b) Box plot showing E2F6 expression levels among different macroscopic types (Polypoid, Ulcerative, and Infiltrative). (c) Box plot comparing E2F6 expression levels between low-grade and high-grade tumors. (d) Survival analysis between high and low E2F6 expression groups.

Kaplan-Meier survival analysis indicated that there was no significant difference in survival rates between the high and low expression groups of E2F6 (Fig. 9).

## Discussion

CRC is a malignancy characterized by high incidence and mortality rates, significantly impacting patients’ quality of life. Our study aims to investigate the role of E2F6 in CRC by integrating high-throughput data analysis with validation from clinical histological samples to comprehensively assess its biological significance in this disease. The results indicate that E2F6 expression is significantly elevated in CRC tissues and is associated with various clinical pathological features. These findings provide crucial evidence for the research of biomarkers in CRC, potentially guiding future directions for personalized treatment.

We utilized a dataset for bioinformatics analysis that encompassed 19 platforms, including mRNA results from 2,449 CRC patients and 1,328 control samples. The analysis revealed that E2F6 mRNA expression levels in CRC tissues were significantly higher than those in non-CRC tissues (AUC = 0.93), consistent with previous studies [13]. Furthermore, we conducted IHC analysis using internal clinical histological samples to validate these findings. Experimental results demonstrated that E2F6 protein levels were significantly elevated in CRC tissues compared to adjacent non-CRC tissues (AUC = 0.91). These results underscore the potential of E2F6 as a valuable diagnostic biomarker for CRC. As CRC continues to be a leading cause of cancer-related deaths globally, the identification of reliable biomarkers is essential for enhancing early detection and customizing therapeutic approaches [25].

E2F6 is significantly associated with cancer development and progression. Numerous studies have shown that abnormal expression of E2F6 can lead to dysregulated cell growth and tumor development, playing a critical role in the progression of various cancers [26–30]. In this study, based on CRISPR gene knockout experiments, we found that E2F6 knockout resulted in a significant decrease in the proliferation of 52 CRC cell lines, indicating that E2F6 promotes CRC cell proliferation. Research has indicated that circDUSP16 can upregulate E2F6 expression by downregulating miR-432-5p, thereby promoting CRC tumor growth [31]. Recent studies suggest that E2F6 may enhance CRC cell proliferation and migration in an Aly/REF output factor (ALYREF)-dependent manner [19]. These findings highlight the critical role of E2F6 in CRC progression and elucidate its potential molecular mechanisms, providing new insights for targeted therapy.

Our study involved 200 CRC patients from the Red Cross Hospital in Yulin, Guangxi, with a follow-up period ranging from 1 to 53 months. Male patients comprised 59.5% of the cohort, while female patients accounted for 40.5%. The ages of the patients ranged from 15 to 92 years, with a mean age of 63 years. Regarding tumor budding, the proportions of BD1, BD2, and BD3 were 55%, 31.5%, and 13.5%, respectively. T-stage results indicated that the proportions of patients in stages I, II, III, and IV were 1.5%, 8.5%, 86.5%, and 3.5%, respectively. In terms of histological grading, the ratios of low-grade to high-grade tumors were 74.5% and 25.5%, respectively. Understanding these patient characteristics is crucial for developing personalized treatment strategies and can aid physicians in making more effective clinical decisions. Our statistical analysis (Table 1) revealed that although the incidence of CRC is generally higher in male patients, female patients exhibited higher levels of E2F6 expression, potentially related to hormonal levels and lifestyle factors. Additionally, CRC patients in the older age group (≥ 60), with high-grade tumor budding, advanced T-stage, and higher histological grades, were more likely to show elevated E2F6 protein expression, indicating a close association between high E2F6 expression and unfavorable prognostic factors. Correlation analysis showed that E2F6 expression was negatively correlated with Ki-67 positivity and positively correlated with pathological type and grade, with all P-values being less than 0.05, indicating statistical significance. These results support the potential of E2F6 as a biomarker for CRC and may assist in guiding clinical decision-making. Although the survival rate difference between high and low expression groups did not reach statistical significance in the Kaplan-Meier survival analysis (P > 0.05), this does not diminish the importance of E2F6 in CRC. Instead, this finding suggests that the role of E2F6 in disease progression warrants further investigation and may provide references for the development of future prognostic biomarkers. These results sharply contrast with previous studies [14, 15, 31, 32, 32–35], which largely did not examine E2F6 in relation to detailed clinicopathological parameters in human cancers—particularly in CRC—thereby highlighting the added value and clinical relevance of our analytical framework. In addition, drug repurposing strategies, such as glucagon-like peptide (GLP)-1–based therapies, and prophylactic targeting of the 20S proteasome with immunomodulatory potential may offer complementary avenues to improve cancer prevention and management [36, 37].

It is essential to recognize the limitations of our study, as they may influence how our findings can be applied. The small sample size and retrospective design might introduce biases that could distort the relationships between E2F6 expression and clinical outcomes. Although our IHC techniques are strong, they do not completely capture the dynamic regulatory mechanisms that affect E2F6 activity in CRC. These limitations raise concerns regarding the generalizability and reliability of E2F6 as a biomarker in various populations. Additionally, the lack of functional validation through laboratory experiments limits our understanding of E2F6’s role in the progression of CRC. To enhance our findings, future research should aim to validate our results in larger, multicentric cohorts and investigate the functional role of E2F6 in CRC development through detailed mechanistic studies. These efforts are crucial for establishing the clinical significance of E2F6 as a biomarker and its potential as a therapeutic target in the ongoing battle against CRC.

In conclusion, this study provides compelling evidence that E2F6 is significantly elevated in CRC tissues and is associated with various clinical factors, underscoring its potential as a novel biomarker for prognosis. The correlation between high levels of E2F6 and adverse prognostic indicators suggests that it could serve as an important marker of tumor aggressiveness. Future research should aim to validate these findings in larger and more diverse patient populations, as well as explore the functional role of E2F6 in cancer biology. By enhancing our understanding of how E2F6 operates, we may pave the way for new treatment strategies and improved outcomes for patients facing CRC.

## Data Availability

The data that support the findings of this study are available from the corresponding author upon reasonable request.

## Abbreviations

AUC: area under the curve
CCLE: Cancer Cell Line Encyclopedia
CI: confidence interval
CRC: colorectal cancer
CRISPR: clustered regularly interspaced short palindromic repeats
dMMR: deficient mismatch repair
E2F6: E2F transcription factor 6
GEO: Gene Expression Omnibus
GTEx: Genotype-Tissue Expression
ICGC: International Cancer Genome Consortium
IHC: immunohistochemistry
MMR: mismatch repair
pMMR: proficient mismatch repair
RNA-seq: RNA sequencing
ROC: receiver operating characteristic
RT-qPCR: reverse transcription quantitative polymerase chain reaction
scRNA-seq: single-cell RNA sequencing
SMD: standardized mean difference
SRA: Sequence Read Archive
sROC: summary receiver operating characteristic
st-seq: spatial transcriptomics sequencing
TCGA: The Cancer Genome Atlas
TNM: tumor-node-metastasis
TPM: transcripts per million
